# Forsythoside A Attenuates High-Fat Diet-Induced Obesity by Regulating Thermogenesis and Browning of White Adipose Tissue Through Activation of the AMPK Signaling Pathway

**DOI:** 10.3390/ph19060852

**Published:** 2026-05-29

**Authors:** Qinyu Meng, Hong Xu, Mengru Zhong, Yuanzhi Mu, Xinyu Zhao, Chenru Lin, Fang Xu, Meizi Yang, Hui Sun, Yingjiang Xu, Yana Li

**Affiliations:** 1 Department of Pathophysiology, School of Basic Medical Sciences, Binzhou Medical University, Yantai 264003, China; 2 The Second Clinical Medical College, Binzhou Medical University, Yantai 264003, Chinalin14233457@outlook.com (C.L.);; 3 The First Clinical Medical College, Binzhou Medical University, Binzhou 256603, China; 13375607061@163.com (Y.M.); black971y@gmail.com (X.Z.)

**Keywords:** obesity, Forsythoside A, AMPK, thermogenesis, browning

## Abstract

**Purpose:** Obesity is a global public health issue, and natural products that promote white fat browning and enhance thermogenesis to consume energy represent promising strategy for addressing this problem. Forsythoside A (FTA) is key bioactive constituent isolated from the fruit of Forsythia suspensa. It has been reported that FTA can alleviate metabolic disorders such as hepatic lipid accumulation induced by high-fat diet (HFD). However, research on the role of FTA in alleviating obesity by promoting white fat browning remains scarce. **Materials and Methods:** We intervened in diet-induced obesity (DIO) mice and differentiated 3T3-L1 cells with FTA and detected thermogenic indices and the expression of thermogenesis-related genes under the guidance of network pharmacology. Mechanistically, molecular docking combined with molecular biology techniques was employed to verify the affinity of pathway-related proteins, and the AMPK inhibitor (BML-275) was used to intervene in 3T3-L1 cells to assist in demonstrating the main pathway through which FTA stimulates white fat browning. **Results:** FTA significantly attenuated lipid accumulation in both in vivo and in vitro models. Gene Ontology (GO) enrichment analysis revealed that FTA may promote white adipocyte browning and mitochondrial thermogenesis. Consistent with improved energy metabolism, FTA treatment increased oxygen consumption and carbon dioxide production in mice, while maintaining the respiratory exchange ratio (RER) at approximately 0.7. In vitro, FTA enhanced cellular oxygen consumption rate (OCR) and mitochondrial density. Kyoto Encyclopedia of Genes and Genomes (KEGG) pathway enrichment analysis combined with molecular docking identified the AMPK signaling cascade as a key potential pathway mediating FTA action. Molecular biology assays further confirmed that FTA promotes AMPK phosphorylation and activates the canonical thermogenic downstream PGC-1α/UCP1 pathway. Consistently, inhibition of AMPK with BML-275 abolished the beneficial effects of FTA in 3T3-L1 adipocytes. **Conclusions:** This study reveals that FTA enhances white fat browning via the AMPK pathway while increasing thermogenesis in adipose tissue.

## 1. Introduction

Obesity is a globally prevalent disease with complex etiology and serves as a risk factor for various conditions, such as diabetes, coronary heart disease, and fatty liver disease [1]. When there is an imbalance between energy intake and energy expenditure, obesity occurs [1]. Systemic energy balance is largely maintained by adipose tissue, whose mass and proportion of body composition directly influence obesity [1,2].

Mammalian adipose tissue is mainly categorized into two types: white adipose tissue (WAT), which is widely considered primarily responsible for energy storage, and brown adipose tissue (BAT), which functions mainly in non-shivering thermogenesis to maintain core body temperature [3]. Adipose tissue is highly plastic and can undergo transformation in response to factors such as sudden temperature changes or hormonal signals [3,4]. Beige adipose tissue arises from the conversion of white adipose tissue [5]. During this process, unilocular, lipid-rich white adipocytes shrink while their mitochondrial density increases, gradually forming beige adipocytes, a process known as “white adipose tissue browning” [6,7]. This “browning” enhances energy expenditure in adipose tissue [6]. A major similarity between beige adipocytes and brown fat is that beige fat also generates heat by highly expressing uncoupling protein 1 (UCP1), which dissipates the proton gradient across the mitochondrial membrane [7]. Even subcutaneous adipocytes, primarily dedicated to energy storage, can transform to prioritize energy expenditure through “browning” [8].

In mammals, white adipose tissue, as a heterogeneous organ, can be divided into two major categories based on anatomical location: subcutaneous adipose tissue (SAT) and visceral adipose tissue (VAT) [7,8,9]. SAT is located beneath the skin, predominantly in the inguinal region, whereas VAT is situated within the abdominal cavity, mainly represented by epididymal adipose tissue (EAT) [9]. Beyond their different locations, EAT and SAT also play distinct roles in regulating energy homeostasis. Thermogenic beige adipocytes are more commonly found in SAT [9,10]. Therefore, targeting “white adipose tissue browning” has become a promising therapeutic approach for treating obesity.

A primary objective of obesity management Is to preserve overall health status [1]. Similar to other chronic medical conditions, the clinical management of obesity necessitates a comprehensive, long-term therapeutic strategy [11]. Prior pharmacological investigations have demonstrated that potent anti-obesity medications can induce greater than 10% body weight reduction in over two-thirds of clinical trial subjects [12]. However, there remain debates over the long-term data regarding their safety, efficacy, and cardiovascular outcomes [12]. To date, there is no definitive pharmaceutical research that can alleviate obesity directly by promoting the browning of WAT.

Traditional Chinese medicine, with its distinct strengths in preventing, treating, and rehabilitating diseases, has contributed significantly to human health and is regarded as a promising natural reservoir for therapeutic drugs [13]. *Forsythiae Fructus* (FF known as “Lianqiao” in traditional Chinese medicine) is the dried mature fruit of Forsythia suspensa (Thunb.) Vahl [14]. *Forsythia* contains more than 200 chemical constituents, mainly including lignans, cyclohexylethanol derivatives, flavonoids, terpenes, phenylethanoid glycosides, and alkaloids. Among them, phenylethanoid glycosides and xylose are the two most abundant components [15,16]. *Forsythiasides* are divided into subtypes A-K based on their nuclear substituent groups. Among them, forsythiaside A and forsythiaside B are the most widely investigated constituents [15]. Forsythoside A (FTA, molecular formula C_29_H_36_O_15_), which possesses notable anti-inflammatory, antioxidant, and hepatoprotective activities, is recognized as the primary bioactive marker constituent of FF [14,17]. Previous studies have demonstrated that FTA can ameliorate hepatic steatosis and reduce the accumulation of free fatty acids in the liver in the context of HFD-induced nonalcoholic fatty liver disease [17,18,19]. However, the role of FTA in obesity and its effect on the conversion of white adipose tissue into beige adipose tissue unexplored.

In this study, we found that FTA could alleviate diet-induced obesity in mice. Notably, mechanistic prediction via network pharmacology indicated that FTA exerts thermogenic and thermogenic gene activation effects. In vivo experiments confirmed that FTA led to increased thermogenesis and browning of white adipose tissue in obese mice. In vitro experiments showed that, with the increase in FTA intervention concentration, both mitochondrial density and UCP1 protein expression in adipocytes were significantly elevated. This study enriches the theoretical basis for natural products in regulating obesity and provides new experimental evidence for the browning of white adipose tissue. It expands the application scope of traditional medicines and offers novel Insights for the clinical treatment of obesity.

## 2. Results

### 2.1. FTA Alleviates Adipose Deposition in DIO Mice

**Figure 1:**
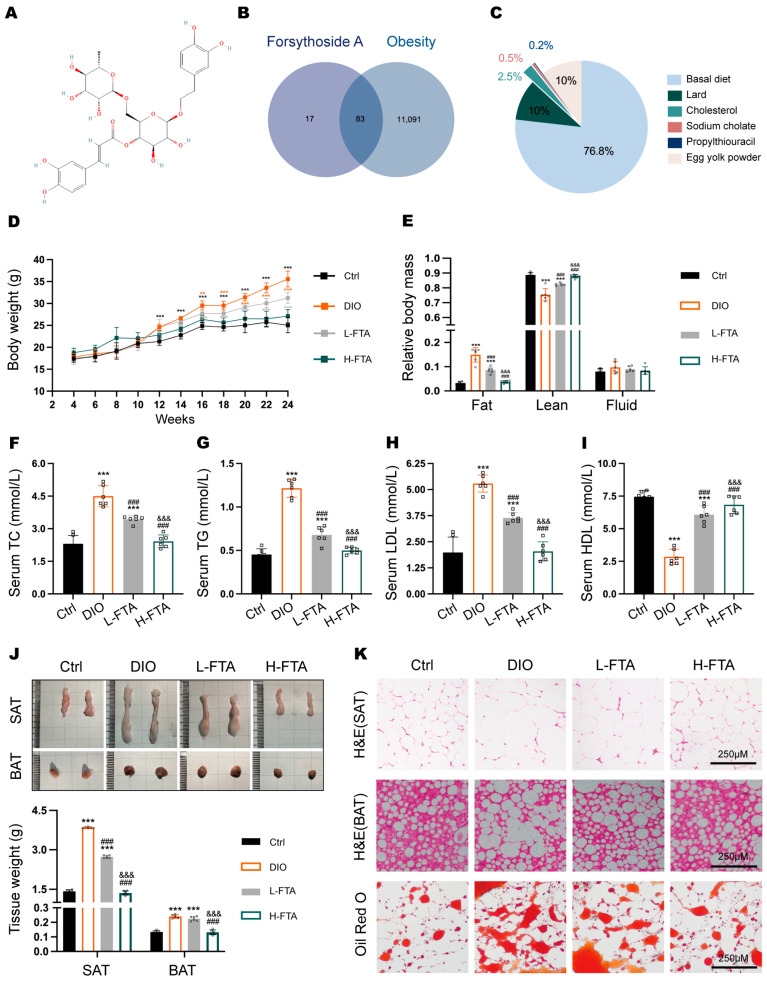
**FTA ameliorates diet-induced obesity in mice.** We identified potential therapeutic targets of FTA and Obesity-related targets, followed by a systematic intersection analysis. (**A**) Formula for the FTA. (**B**) Intersection target. (**C**) Main components of a high-fat diet. During high-fat diet feeding, mice were administered FTA by gavage at low (25 mg/kg) and high (50 mg/kg) doses. (**D**) Body weight (*n* = 6). (**E**) Relative body mass (*n* = 6). (**F**–**I**) Measuring serum TC, TG, LDL, and HDL levels in mice to evaluate lipid metabolism (*n* = 6). (**J**)The weight of subcutaneous fat (SAT) in the groin, and the weight of brown fat (BAT) on the scapula *(n* = 6). (**K**) Representative image of H&E of SAT and BAT, Oil Red O of SAT (scale bar = 250 μm). Data are mean ± SD (at least three independent experiments); ** *p* < 0.01, *** *p* < 0.001, versus the Ctrl group; ^###^ *p* < 0.001, versus the DIO group; ^&&&^ *p* < 0.001, versus the L-FTA group.

We first presented the planar chemical structural formula of FTA (Figure 1A). Based on the Venn diagram analysis, 83 overlapping genes were identified between the potential targets of FTA and obesity-related genes (Figure 1B). The main components of the HFD feed are shown in the pie chart (Figure 1C). During HFD feeding, mice were administered FTA by gavage at low (25 mg/kg) and high (50 mg/kg) doses. It was observed that FTA attenuated the body weight gain induced by the HFD (Figure 1D). Body composition analysis revealed that diet-induced obese mice exhibited increased fat mass and decreased lean mass, which was also mitigated by FTA treatment (Figure 1E). Further analysis of the four serum lipid indicators showed that FTA reduced the levels of TC, TG, and LDL, while increasing HDL levels (Figure 1F–I). Gross morphological examination of adipose tissue indicated that FTA reduced the weight of both inguinal SAT and interscapular BAT (Figure 1G). HE staining demonstrated massive lipid droplet accumulation in both the subcutaneous and BAT of diet-induced obese mice. Oil Red O staining more clearly revealed adipocyte hypertrophy, membrane rupture, and lipid leakage into the intercellular space. These pathological changes, however, were alleviated by FTA in a dose-dependent manner (Figure 1K). The experimental results above indicate that FTA exhibits a significant positive effect in alleviating diet-induced obesity.

### 2.2. Network Pharmacology Reveals the Mechanisms of FTA Ameliorates Diet-Induced Obesity

**Figure 2:**
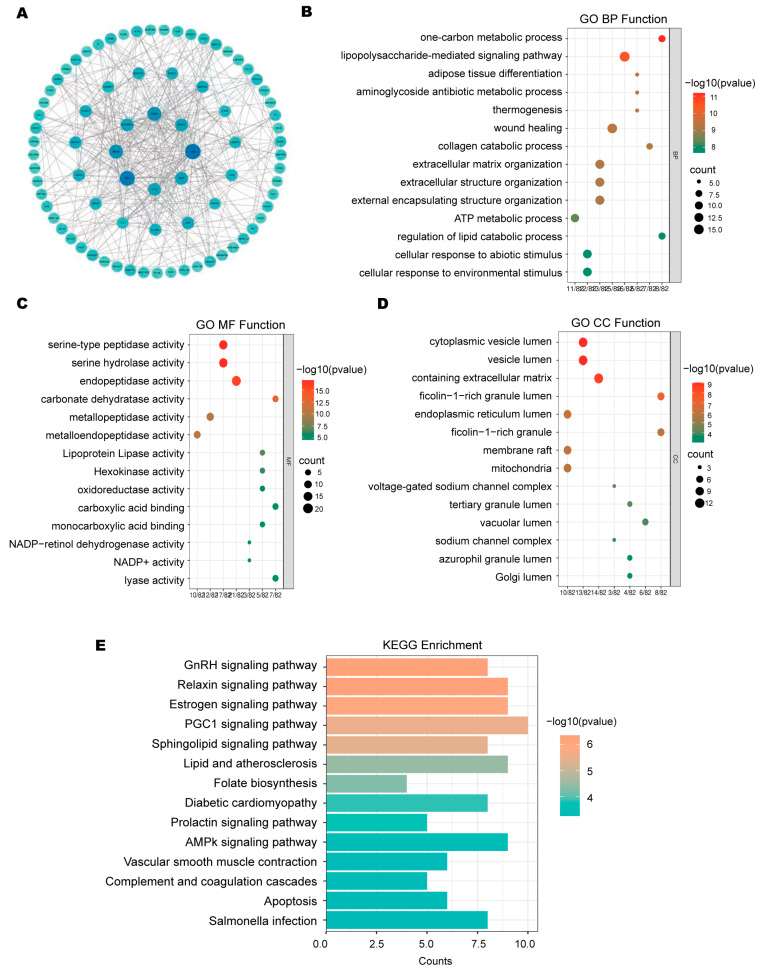
**Network pharmacology reveals the mechanisms of FTA ameliorates diet-induced obesity.** Complete subsequent enrichment analysis on the associated potential targets. (**A**) PPI network diagram of potential targets. (**B**–**D**) GO functional enrichment analysis (BP, MF, and CC). (**E**) Kegg functional enrichment analysis.

This phase of the study aims to investigate the underlying mechanisms by which FTA treats diet-induced obesity. After identifying overlapping genes, we constructed a protein protein interaction (PPI) network (Figure 2A). Gene Ontology (GO) analysis was then performed to examine the molecular functions (MF), biological processes (BP), and cellular components (CC) of these targets. The results revealed enrichment in biological processes related to thermogenesis, adipose tissue differentiation, and ATP metabolism, molecular functions including oxidoreductase activity and NADP+ activity, and cellular components centered on mitochondria (Figure 2B–D). The GO enrichment analysis results indicate that the fundamental mechanisms by which FTA exerts its effects in obesity are associated with thermogenesis, redox processes, and mitochondrial activity. KEGG enrichment analysis further identified the AMPK and PGC1 signaling pathways among the top 15 enriched pathways (Figure 2E). The results of network pharmacology suggest that FTA may treat obesity by influencing WAT browning and adipocyte thermogenesis through the AMPK/PGC1α pathway.

### 2.3. FTA Increases Thermogenesis and Promotes the Browning of WAT in Mice via the AMPK Pathway

**Figure 3:**
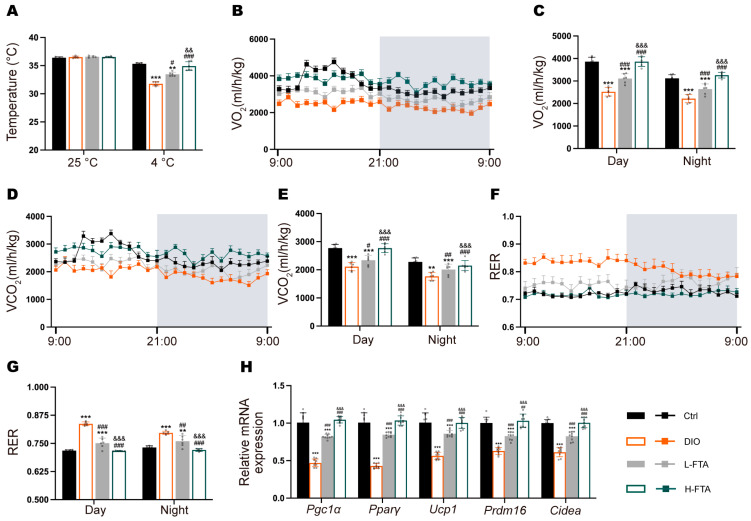
**FTA increases thermogenesis and promotes the browning of WAT in mice via the AMPK pathway.** Guided by the results of network pharmacology, to investigate the effect of FTA on thermogenesis in mice, the mice were subjected to cold exposure at 4 °C. (**A**) Rectal temperature of mice (*n* = 6). (**B**,**C**) Recording and visual display of O_2_ consumption in mice over a 24h circadian cycle (*n* = 6). (**D**,**E**) Recording and visual display of CO_2_ production in mice over a 24h circadian cycle (*n* = 6). (**F**,**G**) The respiratory exchange ratio (RER) of mice over a 24 h circadian period was calculated from the oxygen consumption and carbon dioxide production values (*n* = 6). (**H**) The mRNA expression of thermogenic genes (*Pgc1α*, *Pparγ*, *Ucp1*, *Prdm16*, *Cidea*) (*n* = 9). Data are mean ± SD (at least three independent experiments); ** *p* < 0.01, *** *p* < 0.001, versus the Ctrl group; ^#^ *p* < 0.05, ^##^ *p* < 0.01, ^###^ *p* < 0.001, versus the DIO group; ^&&^ *p* < 0.01, ^&&&^ *p* < 0.001, versus the L-FTA group.

To investigate the effect of FTA on thermogenesis in mice, we first measured their core body temperature. The results clearly indicate that FTA can reduce body heat loss in mice under low-temperature conditions (Figure 3A). To further assess the effect of FTA on energy metabolism in mice in a direct and quantitative manner, the mice were divided into groups and placed in metabolic cages. After a 72 h adaptation period, a continuous 24 h recording of oxygen consumption and carbon dioxide production was conducted. Both oxygen consumption and carbon dioxide production remained at low levels in diet-induced obese mice but were significantly increased after FTA treatment (Figure 3B–E). Calculation of the Respiratory Exchange Ratio revealed that FTA treatment maintained the RER value at approximately 7.0 (Figure 3F,G). Consistent with these findings, FTA treatment increased the expression of these thermogenic genes (*Pgc1α*, *Pparγ*, *Ucp1*, *Prdm16*, *Cidea*) in subcutaneous WAT in a dose-dependent manner (Figure 3H). Based on metabolic cage measurements and relevant mRNA expression data, the findings indicate that FTA primarily utilizes adipose tissue in obese mice as an energy source. This is accompanied by the promoted expression of thermogenic genes in subcutaneous fat, reflecting a browning transformation of WAT.

**Figure 4:**
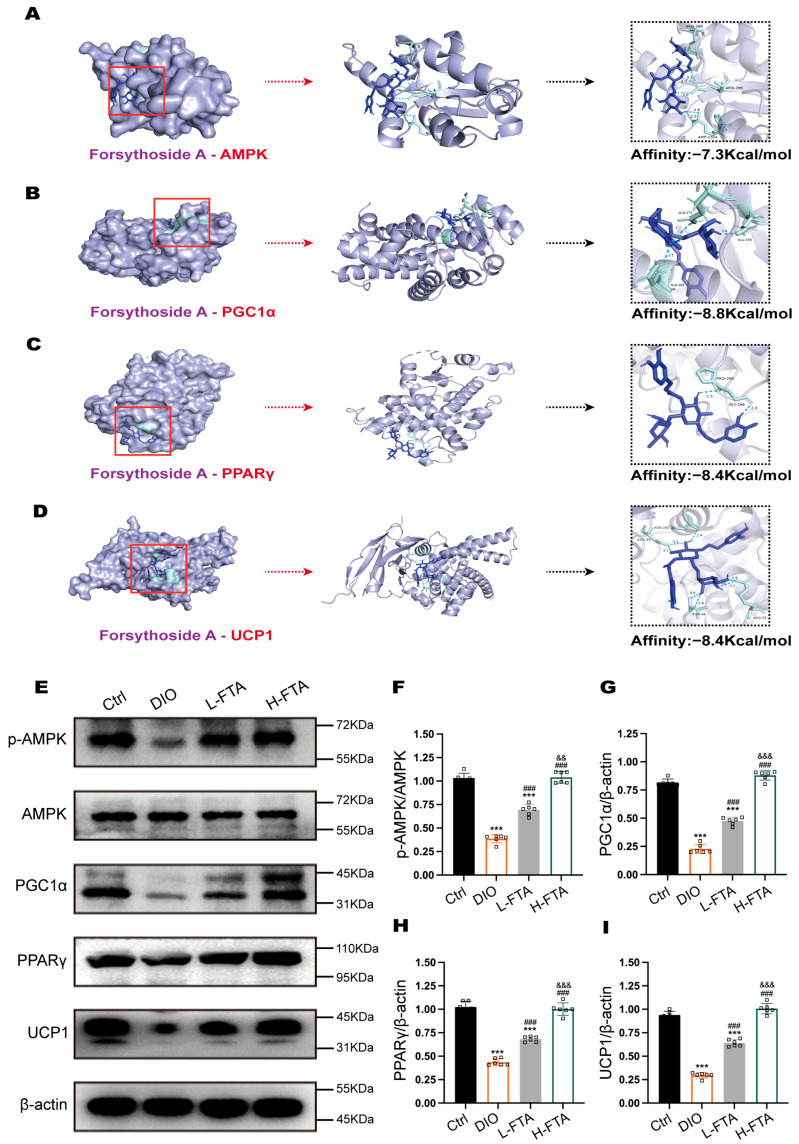
**FTA increases thermogenesis and promotes the browning of WAT in mice via the AMPK pathway.** Mechanistically, we will perform quantitative validation of protein expression based on molecular docking results. (**A**–**D**) Molecular docking simulation of FTA with AMPK, PGC1α, PPARγ, and UCP1. (**E**–**I**) Western blot analysis was conducted to measure the expression levels of P-AMPK/AMPK, PGC1α/β-actin, PPARγ/β-actin, UCP1/β-actin with the corresponding quantitative results shown right (*n* = 6). Data are mean ± SD (at least three independent experiments); *** *p* < 0.001, versus the Ctrl group; ^###^ *p* < 0.001, versus the DIO group; ^&&^ *p* < 0.01, ^&&&^ *p* < 0.001, versus the L-FTA group.

Guided by network pharmacology results indicating the potential efficacy of the AMPK/PGC1 pathway, we performed molecular docking between FTA and key proteins involved in the AMPK-mediated classical thermogenesis pathway. Molecular docking demonstrated that FTA binds with high affinity to AMPK (−7.3 Kcal/mol), PGC1α (−8.8 Kcal/mol), PPARγ (−8.4 Kcal/mol), and UCP1 (−8.4 Kcal/mol) (Figure 4A–D). Western blot analysis confirmed that FTA dose-dependently increased AMPK phosphorylation and upregulated protein levels of PGC1α, PPARγ, and UCP1 in adipose tissue (Figure 4E–I), validating activation of the AMPK-PGC1α-UCP1 thermogenic pathway. Collectively, the above findings provide further evidence that FTA influences adipose tissue energy metabolism and thermogenic gene expression through activation of the AMPK signaling pathway.

### 2.4. FTA Increases Cellular Thermogenesis via AMPK in 3T3-L1 Cells

**Figure 5:**
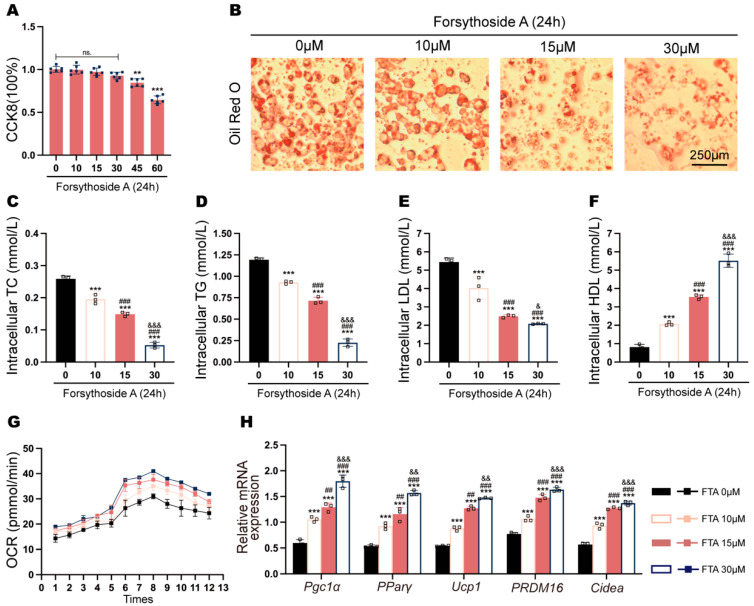
**FTA increases cellular thermogenesis via AMPK in 3T3-L1 cells.** In vitro experiments, we chose to use the classical method to induce adipogenesis in 3T3-L1 cells. (**A**) The cell viability was detected after FTA intervention (*n* = 6). (**B**) Representative image of Oil Red O of 3T3-L1 cells (scale bar = 250 μm). (**C**–**F**) Measuring intracellular TC, TG, LDL, and HDL levels in mice to evaluate lipid metabolism (*n* = 3). (**G**) Oxygen consumption rate (OCR) Assay of 3T3-L1 cells (*n* = 3). (**H**) The mRNA expression of thermogenic genes (*Pgc1α*, *Pparγ*, *Ucp1*, *Prdm16*, *Cidea*) (*n* = 3). Data are mean ± SD (at least three independent experiments); ** *p* < 0.01, *** *p* < 0.001, versus the FTA = 0 μM group; ^##^ *p* < 0.01, ^###^ *p* < 0.001, versus the FTA = 10 μM group; ^&^ *p* < 0.05, ^&&^ *p* < 0.01, ^&&&^ *p* < 0.001, versus the FTA = 15 μM group; n.s. indicates no statistical significance.

For the in vitro experiments, we assessed the impact of FTA on thermogenesis in 3T3-L1 cells. FTA treatment (24 h) showed no significant cytotoxicity at concentrations up to 60 μM (Figure 5A). Oil Red O staining showed that intracellular lipid accumulation decreased in a dose-dependent manner, paralleled by reduced TC, TG, and LDL, but increased HDL levels (Figure 5B–F). In addition, the increase in cellular oxygen consumption induced by FTA correlated with the administered concentration (Figure 5G). We also measured the expression of thermogenic genes (*Pgc1α*, *Pparγ*, *Ucp1*, *Prdm16*, *Cidea*) within cells and found that FTA likewise increased their expression levels (Figure 5H).

**Figure 6:**
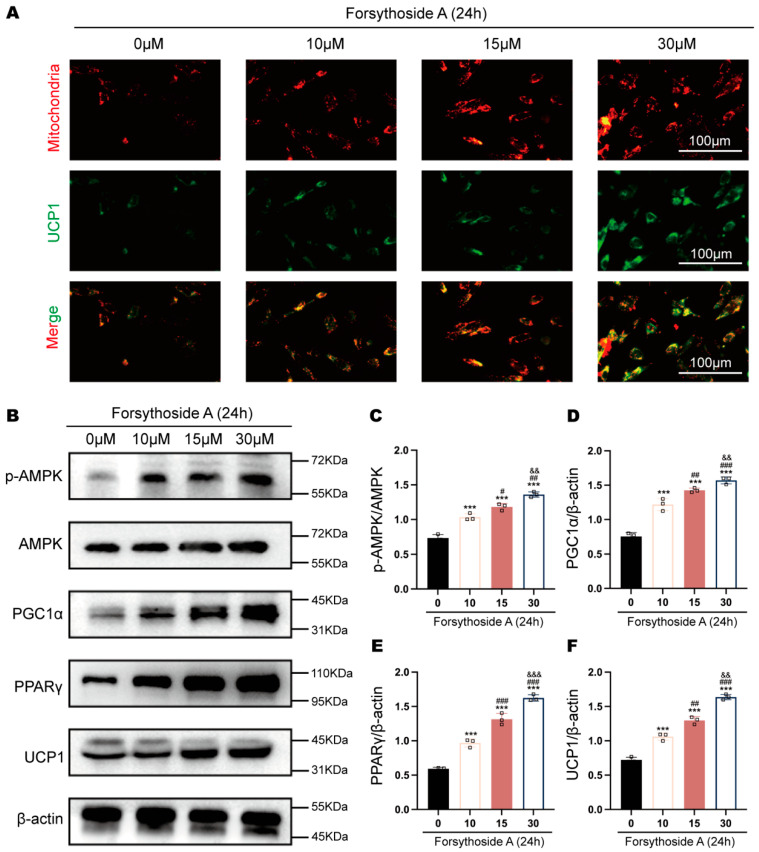
**FTA increases cellular thermogenesis via AMPK in 3T3-L1 cells.** (**A**) Mitochondrial density in 3T3-L1 cells was observed by red fluorescence, and UCP1 protein expression was observed by green fluorescence (scale bar = 100 μm). (**B**–**F**) Western blot analysis was conducted to measure the expression levels of P-AMPK/AMPK, PGC1α/β-actin, PPARγ/β-actin, UCP1/β-actin, with the corresponding quantitative results shown below (*n* = 3). Data are mean ± SD (at least three independent experiments); *** *p* < 0.001, versus the FTA = 0 μM group; ^#^ *p* < 0.05, ^##^ *p* < 0.01, ^###^ *p* < 0.001, versus the FTA = 10 μM group; ^&&^ *p* < 0.01, ^&&&^ *p* < 0.001, versus the FTA = 15 μM group.

Concurrently, fluorescent labeling showed increases in both mitochondrial density (red fluorescence) and UCP1 protein expression (green fluorescence) following FTA treatment (Figure 6A). Western blot analysis confirmed dose-dependent increases in AMPK phosphorylation, and protein expression of PGC1α, PPARγ, and UCP1 (Figure 6B–F). In vitro results further support that FTA reduces adipogenic induction in 3T3-L1 cells and enhances cellular thermogenesis via the AMPK pathway.

**Figure 7:**
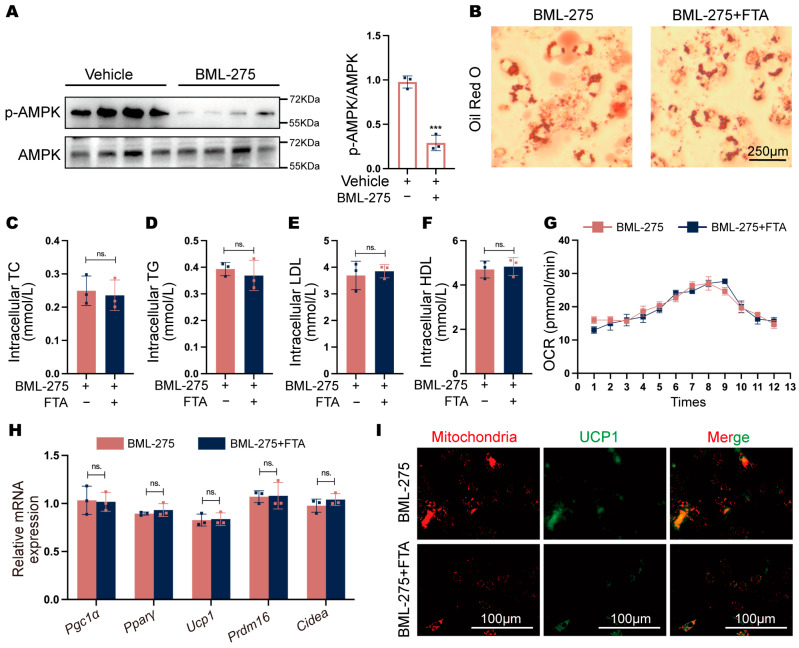
**FTA increases cellular thermogenesis via AMPK in 3T3-L1 cells.** To confirm that FTA acts through the AMPK pathway, we treated adipocytes with the AMPK inhibitor BML-275. Cells were exposed to BML-275 10 μM for 24 h. (**A**) Western blot analysis was performed to examine the expression of P-AMPK in 3T3-L1 cells under BML-275 treatment (*n* = 3). (**B**) Representative image of Oil Red O of 3T3-L1 cells (scale bar = 250 μm). (**C**–**F**) Measuring intracellular TC, TG, LDL, and HDL levels in mice to evaluate lipid metabolism (*n* = 3). (**G**) Oxygen consumption rate (OCR) Assay of 3T3-L1 cells (*n* = 3). (**H**) The mRNA expression of thermogenic genes (*Pgc1α*, *Pparγ*, *Ucp1*, *Prdm16*, *Cidea*) (*n* = 3). (**I**) Mitochondrial density in 3T3-L1 cells was observed by red fluorescence, and UCP1 protein expression was observed by green fluorescence (scale bar = 100 μm). Data are mean ± SD (at least three independent experiments); *** *p* < 0.001, versus the Vehicle group; n.s. indicates no statistical significance.

To confirm that FTA acts through the AMPK pathway, we treated adipocytes with the AMPK inhibitor BML-275. Cells were exposed to FTA at working concentrations of 30 μM, while the BML-275-treated group received 10 μM BML-275 24 h. Western blotting results revealed a significant decrease in the expression of phosphorylated AMPK (p-AMPK) protein in the cells (Figure 7A). Oil Red O staining and lipid profiling indicated that the regulatory effects of FTA on intracellular lipid accumulation and on the levels of TC, TG, LDL, and HDL were blocked by BML-275 (Figure 7B–F). Similarly, when cells were exposed to BML-275, no increase in oxygen consumption was observed upon FTA treatment (Figure 7G). BML-275 abolished the ability of FTA to upregulate specific thermogenesis-related genes (Figure 7H). Fluorescence staining further confirmed that there was no increase in mitochondrial mass and UCP1 expression by FTA, which was attenuated by BML-275 (Figure 7I). These results indicate that FTA promotes thermogenesis and inhibits adipogenesis in an AMPK-dependent manner.

### 2.5. FTA Reduces Pre-Established Obesity in Mice

**Figure 8:**
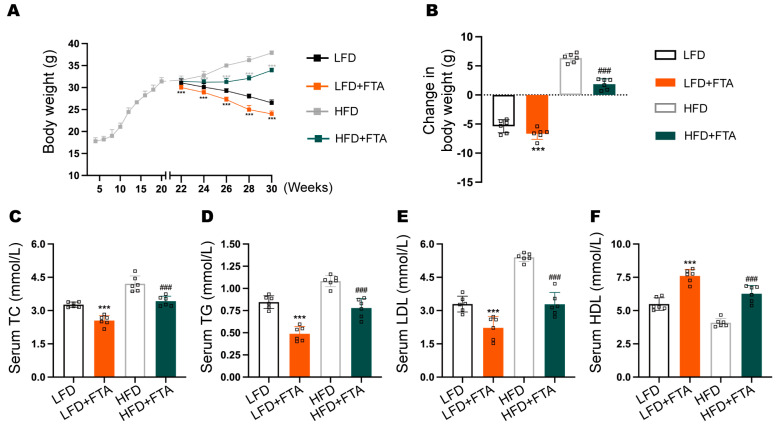
**FTA reduces pre-established obesity in mice.** In this part, after high-fat feeding, obese mice were subjected to a low-fat diet or a continued high-fat diet, combined with FTA drug treatment. (**A**) Body weight (*n* = 6). (**B**) Change in body weight (*n* = 6). (**C**–**F**) Measuring serum TC, TG, LDL, and HDL levels in mice to evaluate lipid metabolism (*n* = 6). Data are mean ± SD (at least three independent experiments); *** *p* < 0.001, versus the LFD group; ^###^ *p* < 0.001, versus the HFD group.

In this final phase of the research, we aim to observe whether FTA’s approach to alleviating obesity remains effective after obesity has already been established. After feeding mice an HFD for 20 weeks, a portion of the mice were switched to an LFD. Meanwhile, some mice on both the low-fat and HFD were administered FTA by gavage at a dose of 50 mg/kg. We found that resuming an LFD combined with FTA treatment reduced body weight; even FTA treatment alone also resulted in weight loss (Figure 8A,B). This ameliorative effect was also observed in serum lipid profiles. Even in the absence of an LFD, FTA similarly reduced the levels of TC, TG, and LDL, while increasing those of HDL (Figure 8C–F). Collectively, these findings indicate that FTA remains effective in mitigating symptoms in mice with pre-existing obesity.

## 3. Discussion

The induction of WAT browning and enhancement of adipose thermogenesis are currently recognized as a robust approach for mitigating obesity [20]. The AMPK signaling pathway, a core regulator of energy metabolism balance, plays a crucial role in both the development and treatment of obesity. Natural products are emerging as highly promising candidates for future therapies of chronic metabolic diseases, due to their favorable safety and druggability. In this work, network pharmacology-guided analysis identified FTA as a potent inducer of white adipocyte browning, which also upregulated key thermogenic genes. Subsequent in vitro and in vivo experiments further validated that the biological actions of FTA depend, at least in part, on activation of the AMPK pathway. Thus, this study establishes that FTA exerts anti-obesity effects by promoting WAT browning through AMPK signaling.

Obesity is a complex disease with a profound impact on public health worldwide, and its prevalence is on the rise [20]. It arises from the interplay of multiple complex factors in individuals, including genetic susceptibility, diet, metabolism, and physical activity [1,21]. Despite the availability of a wide range of surgical and pharmacological interventions, there remains no risk-free and effective therapy for weight management [1]. Lifestyle modifications, dietary changes, and reduced sedentary behavior are currently recognized as the optimal options [1,22]. Given the systemic effects of pharmaceutical use and its associated side effects, natural products represent an effective choice as an anti-obesity agent [23]. Prior research has established Banxia Baizhu Tianma Decoction as an effective intervention for obesity. Additionally, flavonoid-rich lotus leaf extracts have been found to reduce adiposity in mice fed an HFD, acting via gut microbiota-dependent regulation of BAT thermogenesis [24,25]. The flavonoid eriodictyol has also been reported to induce white fat browning in obese mice under HFD conditions [26]. Complementing these preclinical findings, clinical studies have documented anti-obesity effects for several natural agents, such as coffee, tea tree preparations, the green alga *Caulerpa acemose*, garlic, and a combination of ephedra, coix seed, corktree bark, and licorice [27]. Taken together, these data highlight the clear therapeutic potential of plant extracts in addressing obesity.

FTA is a phenylethanol glycoside isolated from air-dried fruits of Forsythia [14]. A large body of experimental research has explored the pharmacological characteristics of FTA. Accumulating evidence demonstrates that FTA exerts favorable therapeutic effects on inflammatory disorders, oxidative damage, and hepatic damage by modulating diverse cellular signaling pathways [17]. In vivo experiments of chronic disease models, the therapeutic dose of FTA for alleviating osteoarthritis was 30 mg/kg via intragastric administration every other day for 8 weeks [28]. For the treatment of pulmonary fibrosis, the low-dose group of FTA was given 20 mg/kg via continuous intragastric administration for 21 days, and the high-dose group was given 40 mg/kg [29]. Based on these findings and the body weight monitoring results of obese mice induced by an HFD during the experiment, we determined the therapeutic doses of FTA as follows: 25 mg/kg for the low-dose group and 50 mg/kg for the high-dose group (Figure 1). In vitro experiments, referring to the applied doses of FTA in HUVECs and HFL1 cells (10–30 μM) and combining the results of cell viability assays, three different doses were selected for subsequent experiments (Figure 5A). In studies on the hepatoprotective effects of FTA, it has been found that FTA can reduce body weight gain and hepatic lipid accumulation in HFD-fed mice, exhibiting certain anti-lipid metabolic disorder activity [19]. Consistently, our findings demonstrate that FTA can also alleviate body weight gain and adipose tissue hypertrophy in HFD-fed mice (Figure 1B,H).

In mammals, WAT functions not merely as a site for energy storage, but also plays key roles in endocrine and paracrine signaling. This tissue exhibits remarkable plasticity [30]. Furthermore, WAT can differentiate into beige adipose tissue, a process known as adaptive thermogenesis [30]. Similar to BAT, beige adipose tissue displays adaptive features such as upregulated UCP1 expression, elevated adipocyte density, and increased mitochondrial abundance [31]. Extensive research into the anti-obesity properties of natural products has shown that phytochemicals exert their beneficial effects via multiple pathways. These include inhibiting digestive enzymes (e.g., pancreatic lipase and amylase), modulating appetite, suppressing WAT formation, or stimulating WAT browning [32,33]. Certain bioactive compounds derived from fruits, vegetables, and edible plants—such as curcumin from turmeric and nobiletin from citrus peels—have been found to promote thermogenesis following the browning of WAT [34,35]. The browning of WAT induces adaptive thermogenesis, which is essential for homeotherms [36]. Our results demonstrated that FTA enhances thermogenesis in obese mice to maintain normal body temperature upon cold stimulation (Figure 3A). Furthermore, organismal thermogenesis primarily stems from the aerobic oxidation of macronutrients, a process that consumes oxygen and produces carbon dioxide [36,37]. Consistently, FTA increased O_2_ consumption and CO_2_ production, and maintained the respiratory exchange ratio (RER) at approximately 0.7 (RER ≈ 0.7 indicates that fat is the primary fuel source for energy metabolism) (Figure 3B–G). At the genetic level, it is currently widely accepted that the distinction between beige adipocytes and white adipocytes can be made by detecting the expression of UCP1 and PR domain-containing protein 16 (PRDM16) [38]. In addition, cell death-inducing DFFA-like effector A (Cidea), a protein highly abundant in BAT, can enhance the binding of peroxisome proliferator-activated receptor γ (PPARγ) to the UCP1 promoter, thus driving UCP1 transcriptional upregulation [39,40]. Previous studies have shown that natural products such as actein, garlic scape, and berberine can upregulate the expression of the genes in WAT and cells during the induction of white fat browning [41,42,43]. The present study’s findings align with these earlier reports (Figure 3H and Figure 5H).

AMP-activated protein kinase (AMPK) serves as a core modulator of energy homeostasis, orchestrating multiple metabolic processes to maintain a proper balance between nutrient availability and energy consumption [44]. As an evolutionarily conserved serine/threonine kinase, AMPK integrates metabolic signals from both peripheral tissues and the central nervous system to modulate whole-body energy homeostasis [45]. Studies have demonstrated that AMPK in specific regions of the body can mediate the thermogenic actions of various circulating signals (e.g., thyroid hormone, GLP-1, estradiol) in a food intake-independent manner [45,46,47]. The link between AMPK activation and thermogenic fat operates via two distinct pathways: downregulation of AMPK in the hypothalamus or activation of AMPK within adipocytes [48]. Among natural products reported to drive WAT browning, hyperforin has been shown to trigger adipose thermogenesis via the Dlat-AMPK axis, thereby mitigating obesity [49]. In addition, *Panax notoginseng* saponins modulate the gut microbiota and promote both thermogenesis and beige adipocyte remodeling in diet-induced obesity models through the leptin-dependent AMPK/STAT3 pathway [50]. In studies on the effects of FTA on the AMPK pathway, FTA exerts an activating effect on the AMPK pathway and alleviates inflammatory injury in MAC-T cells via the AMPK/mTOR pathway [51]. In hepatic oxidative stress injury, FTA also exerts a mitigating effect by activating the AMPK pathway through Nrf2 [52]. The molecular docking model constructed in this study also clearly revealed a significant affinity between FTA and AMPK; moreover, the upregulatory effect of FTA on AMPK has been verified in both in vivo and in vitro samples (Figure 4 and Figure 6).

Furthermore, the thermogenic potential of thermogenic adipose tissue is closely linked to its mitochondrial content, and accumulating research underscores AMPK’s critical role in sustaining mitochondrial function [53]. Our study also observed that FTA could increase mitochondrial density in adipocytes (Figure 6A). BML-275, a widely used AMPK inhibitor, suppresses both baseline and 2DG-stimulated AMPK phosphorylation in HT1080 cells [54]. In our in vitro assays, we confirmed that the thermogenic effects of FTA in adipocytes, including enhanced white fat browning, are mediated by AMPK activation. Consistently, BML-275 treatment eliminated these effects, including the FTA-driven increase in mitochondrial density (Figure 7).

In conclusion, regarding the treatment of obesity, natural products stand out for their advantage of minor side effects without resorting to invasive and damaging approaches such as surgery [12]. In basic research, the administration of natural products to small animals is mainly carried out synchronously with the establishment of small animal models, whereas in clinical application, drugs usually exert their effects after the onset of diseases [55,56]. In our study, after constructing the DIO mouse model, we restored the mice to an LFD and treated them with FTA, and also observed a significant effect of FTA in alleviating obesity-related symptoms (Figure 8). This finding suggests that FTA possesses certain therapeutic potential for the treatment of established obesity.

Additionally, FTA may cause potential side effects at high doses. Excessive FTA exposure can induce cytotoxicity, oxidative stress injury, and inflammatory responses in normal cells. High concentrations of FTA also alter cell morphology, suppress normal cell proliferation, and disrupt intracellular calcium homeostasis and related signaling pathways. Accordingly, the dose and administration of FTA should be strictly controlled in pharmacological studies and practical applications to prevent adverse biological effects.

## 4. Materials and Methods

### 4.1. Chemistry

Forsythoside A (FTA, purity > 98%) and the AMPK inhibitor BML-275 (purity = 98.96%) were commercially acquired from MedChemExpress Biotechnology Co., Ltd. (South Brunswick, NJ, USA). Insulin utilized in this experiment was sourced from Beyotime Biotechnology Co., Ltd. (Shanghai, China). Dexamethasone and 3-isobutyl-1-methylxanthine (IBMX) were purchased from Sigma-Aldrich Biotechnology Co., Ltd. (St. Louis, MO, USA).

### 4.2. Animal Protocol

#### 4.2.1. General Conditions and Grouping of Experimental Animals

C57BL/6 mice used in this study were purchased from Jinan PengYue Experimental Animal Breeding Co., Ltd. (Jinan, China). All animals were raised in SPF-level laboratory animal rooms. The feeding environment was maintained at a constant temperature of (20 ± 2) °C, relative humidity ranging from 40% to 70%, and a 12 h light-dark cycle. Before formal experiments, all mice were adaptively raised for one week with free access to a standard diet and drinking water. The whole animal experimental protocol was reviewed and approved by the Animal Ethics Committee of Binzhou Medical University [57]. Mice were randomly grouped and subjected to corresponding drug interventions, 6 experimental animals per group: (1) Ctrl: Mice received interventions with saline. (2) DIO: Mice were fed an HFD for 20 weeks beginning from the fourth week. (3) L-FTA: Mice were administered FTA by gavage (25 mg/kg) for 4 consecutive weeks. (4) H-FTA: Mice were administered FTA by gavage (50 mg/kg) for 4 consecutive weeks. The body weight of the mice was measured every 7 days to observe the changes.

In the final part of this study, mice fed with HFD reaching over 30 g with higher body fat percentage were selected and randomly grouped: (1) LFD: Mice were fed a low-fat diet (LFD). (2) LFD + FTA: Mice were fed an HFD and administered FTA by gavage (50 mg/kg) for 4 consecutive weeks. (3) HFD: Mice were fed an HFD. (4) HFD + FTA: Mice were fed an HFD and administered FTA by gavage (50 mg/kg) for 4 consecutive weeks.

#### 4.2.2. The Formula of the HFD

The HFD was provided by Jinan Pengyue Laboratory Animal Technology Co., Ltd. (Jinan, China). The HFD is composed of 76.8% basal feed, 10% lard, 10% egg yolk powder, 2.5% cholesterol, 0.5% sodium cholate, and 0.2% propylthiouracil, formulated to induce hyperlipidemia or related metabolic conditions in experimental animals.

### 4.3. Body Fat Rate and Organ Ratio

Body composition analysis was performed on 20-week-old mice from all experimental groups. Under ambient conditions (20–25 °C), fat, lean tissue, and fluid mass were quantified using a dedicated body fat monitor (Bruker BioSpin GmbH, Rheinstetten, Germany) [57].

### 4.4. Biochemical Measurement of Lipids

The levels of serum lipid parameters were detected in mouse serum and 3T3-L1 cell homogenates. The lipid metabolism kits were provided by Nanjing Jiancheng Biotechnology Co., Ltd. (Nanjing, China). The entire experimental procedure was carried out strictly following the reagent manufacturer’s standard operating protocols [58].

### 4.5. H&E Staining

Refer to the manual of the hematoxylin-eosin staining kit to stain paraffin sections of adipose tissue. The kit was supplied by Solarbio Science & Technology Co., Ltd. (Beijing, China) [50].

### 4.6. Oil Red O Staining

Fat frozen sections and 3T3-L1 cells were processed following the operating protocol of the Oil Red O staining kit. The kit was supplied by Solarbio Science & Technology Co., Ltd. (Beijing, China) [57].

### 4.7. Network Pharmacology

#### 4.7.1. The Collection of Targets

The chemical structure of FTA was obtained from the PubChem database https://pubchem.ncbi.nlm.nih.gov/ (accessed on 22 March 2025) in the form of its SMILES notation. Potential molecular targets of FTA were then predicted using the SwissTargetPrediction online platform https://www.swisstargetprediction.ch/ (accessed on 23 March 2025). Following data integration and removal of duplicate entries, a set of candidate targets for FTA was established. To identify obesity-related targets, the GeneCards database https://www.genecards.org/ (accessed on 23 March 2025) was searched using the keyword “Obesity.” Only targets with a relevance score of ≥15 retained for subsequent analysis [57].

#### 4.7.2. Enrichment Analysis

With the support of the Microbial Informatics platform http://www.bioinformatics.com.cn/login/ (accessed on 30 March 2025), the clusterProfiler package (version 4.0) was adopted to conduct enrichment analysis. GO functional annotation and KEGG pathway enrichment were carried out for the intersecting targets. Afterwards, the top 15 terms with significant GO enrichment and the top 15 enriched KEGG pathways were screened out, and then presented in the form of bar charts [57].

#### 4.7.3. Associated Targets PPI Network

Drug-disease intersecting targets were uploaded to the STRING database https://cn.string-db.org/ (accessed on 4 September 2025). The minimum confidence score was set to 0.7, and isolated nodes were removed from the analysis. The resulting protein–protein interaction (PPI) data were downloaded in TSV format and subsequently imported into Cytoscape 3.8.1 for network construction. The topological properties of the established network were then assessed using the Network Analyzer plugin [59].

### 4.8. Molecular Docking

Molecular docking was performed using PyMOL3.1 and AutoDock4 (v4.2.6). The 2D structure of FTA was drawn in ChemDraw 18.0 and optimized with the MM2 force field. 3D structures of target proteins (AMPK: ARER; PGC1α: 1XB7; PPARγ: 1PGR; UCP1: 8HBN) were acquired from the PubChem database. These protein structures were processed in PyMOL3.1 to remove water, original ligands, and other non-protein elements. AutoDock4 was then used to conduct the docking simulations, applying the default genetic algorithm for calculations under consistent conditions. Analysis and evaluation of the docking results were carried out using the tools provided within AutoDock [59].

### 4.9. Energy Metabolism Index

In energy metabolic research, indirect calorimetry was applied to assess metabolic status in mice using CLAMS-8 (Columbus Instruments, Columbus, OH, USA). This system automatically recorded relevant data, including oxygen consumption (VO2), carbon dioxide release (VCO2), and respiratory exchange ratio (RER). Each mouse was placed separately into a metabolic chamber and allowed unlimited access to a standard diet and drinking water. The gas pathway was connected and detection was performed. After a 24 h acclimation period, continuous metabolic data were collected for 24 h and analyzed using the Comprehensive Lab Animal Monitoring System (CLAX) for data acquisition and analysis [42].

### 4.10. Cell Culture and Differentiation

The 3T3-L1 cell line was obtained from Procell Life Science & Technology Co.,Ltd. Cells were maintained in DMEM supplemented with 10% NCS and 100 μg/mL penicillin-streptomycin, at 37 °C in a humidified 5% CO_2_ incubator. When cells reached adequate confluence, the culture medium was switched to 10% FBS to synchronize the cell cycle. Differentiation was then initiated by culturing cells in induction medium containing 10 μg/mL insulin, 0.25 μM dexamethasone, and 0.5 mM IBMX. After 48 h, cells were transferred to maturation medium supplemented with 10% FBS and 10 μg/mL insulin. On day 4 of differentiation, the medium was replaced with complete culture medium lacking insulin. The evaluation was ultimately performed via Oil Red O staining [60].

### 4.11. Oxygen Consumption Rate (OCR) Assay

Following the protocol instructions, differentiated 3T3-L1 cells under sealed conditions were treated with an OCR fluorescent probe. The detection wavelength settings were adjusted according to the instructions of the Enhanced Oxygen Consumption Rate Assay Kit, which was obtained from Elabscience Biotechnology Co., Ltd. (Wuhan, China). [61].

### 4.12. UCP1 Immunofluorescence and Mitochondrial Staining

Coverslips were placed in 12-well plates before seeding cells. After 24 h of adherence, cells were rinsed with PBS, fixed in 4% paraformaldehyde, and permeabilized using 0.25% Triton X-100 (Thermo Fisher, Waltham, MA, USA). Cells were then washed three times with PBS and blocked with 5% BSA for 30 min. Samples were incubated overnight at 4 °C with anti-UCP1 primary antibody (1:300), washed three times, and then labeled with FITC-conjugated goat anti-rabbit secondary antibody (1:500). Images were captured on a DMi8 inverted fluorescence microscope and analyzed via Leica Application Suite X software v5.3.1 (LAS X).

For mitochondrial labeling, cells were incubated with 200 nM Mito-Tracker Red CMXRos (prepared from a 200 μM stock) at 37 °C for 15–30 min. Mito-Tracker Red was sourced from Thermofisher Biotechnology Co., Ltd. (Waltham, MA, USA). After staining, cells were fixed in 4% paraformaldehyde, rinsed with PBS, and subjected to immunostaining as described above [62].

### 4.13. Western Blot

Proteins were extracted from subcutaneous adipose tissue and 3T3-L1 cells using RIPA lysis buffer (Beyotime, Shanghai, China) supplemented with 1% PMSF (Beyotime, Shanghai, China) and phosphatase inhibitors. After mixing with 5× loading buffer, the samples were boiled for 10 min. The proteins were separated by SDS-PAGE (Epizyme, Shanghai, China) and electrotransferred onto PVDF membranes (Merck KGaA, Darmstadt, Germany). Post blocking with skim milk, primary antibodies were applied to the membranes for overnight incubation. Following TBST (Epizyme, Shanghai, China) washes, blots were probed with secondary antibodies. Bands were visualized by ECL using the Tanon 5200 imaging system. Antibody details and dilutions are provided in Table 1.

### 4.14. qRT-PCR

Total RNA was isolated from subcutaneous adipose tissues and 3T3-L1 cells with the SteadyPure Rapid RNA Extraction Kit (Accurate, Guangzhou, China). A UV spectrophotometer manufactured by Thermo Fisher was adopted to detect RNA concentration and ensure the quality for subsequent reverse transcription. Genomic DNA elimination was performed with 4× *g* DNA Wiper Mix (Vazyme, Nanjing, China), RNA template, and DEPC water (Biosharp, Hefei, China), under the condition of 42 °C for 2 min. Reverse transcription was performed using 5 × HiScript II qRT SuperMix IIa (Vazyme, Nanjing, China) under the following conditions: 37 °C for 15 min, followed by 85 °C for 5 s, on a Bio-Rad T100 Thermal Cycler. For qRT-PCR, 2 μL of cDNA template was used per sample. The reaction included an initial pre-denaturation step, followed by 40 cycles of denaturation, annealing, and extension. Amplification was carried out on a LightCycler96 system. Relative gene expression was calculated using the 2^−^ΔΔCt method. All primer sequences are listed in Table 2.

### 4.15. Statistical Analysis

Statistical processing was carried out using SPSS 20.0 (IBM, USA). Data are expressed as the mean ± standard deviation (SD). Pearson correlation analysis was used to examine associations between normally distributed variables. Student’s *t*-test was applied for two-group comparisons, and one-way ANOVA for multi-group comparisons. Post hoc tests included LSD (equal variances) and Tamhane’s test (unequal variances). All assays were performed in three independent biological replicates, with three technical replicates per sample to guarantee reproducibility and reliability. Statistical significance was defined as *p* < 0.05.

## 5. Conclusions

In this study, FTA alleviated diet-induced obesity in mice. Network pharmacology predicted that FTA promotes thermogenesis and activates thermogenic genes. HFD successfully induced typical obese phenotypes in DIO mice, including increased body weight, adipose tissue mass, serum triglyceride, and cholesterol levels. Meanwhile, SAT and BAT weight increased, and H&E staining showed obvious lipid droplet accumulation. FTA treatment markedly reversed these obese manifestations. FTA exerted prominent thermogenic effects in adipose tissue, as evidenced by improved respiratory metabolism, increased mitochondrial density, and upregulated thermogenic gene expression. In vitro, elevated FTA concentration significantly enhanced mitochondrial density and UCP1 expression in adipocytes. Further verification with BML-275 confirmed that FTA enhances thermogenesis via the AMPK signaling pathway. This work provides a theoretical basis for natural products in obesity intervention, offers new evidence for white adipose tissue browning, expands the application value of traditional herbal components, and provides novel insights for obesity prevention and clinical treatment.

## Figures and Tables

**Table 1:** The antibody used for the Western blot.

Antibody	Dilution	Source	Company
AMPK	1:1000	Rabbit	Proteintech (Rosemont, IL, USA)
P-AMPK	1:1000	Rabbit	Proteintech
PGC-1α	1:1000	Rabbit	Proteintech
PPARγ	1:1000	Mouse	Proteintech
UCP1	1:500	Mouse	Proteintech
β-actin	1:2000	Mouse	Proteintech
Anti-Mouse IgG	1:5000	Goat	Abcam (Cambridge, UK)
Anti-Rabbit IgG	1:5000	Goat	Abcam

**Table 2:** The sequence used for real-time quantitative PCR.

Cene	Forward	Reverse
PParγ	CCTCTCCGTGATGGAAGACC1	CCATTGGGTCAGCTCTTGTG
PRDM16	GATGGGAGATGCTGACGGAT	TGATCTGACACATGGCGAG
Cidea	CGGGAATAGCCAGAGTCACC1	TGTGCATCGGATGTCGTAGG
PGC1α	ATGTGTCGCCTTCTTGCTCT	ATCTACTGCCTGGGGACC1TT
UCP1	AATCAGCTTTGCTTCCCTCA	GCTTTGTGCTTGCATTCTGA

## Data Availability

The original contributions presented in this study are included in the article. Further inquiries can be directed to the corresponding authors.

## Abbreviations

**FTA**, Forsythoside A; **DIO**, diet-induced obesity; **AMPK**, AMP-activated protein kinase; **PGC1α**, Peroxisome proliferator activated receptor γ coactivator-1α; **UCP1**, Uncoupling protein 1; **PPARγ**, Peroxisome proliferative activated receptor γ; **Cidea**, Cell Death-Inducing DNA Fragmentation Factor Alpha-like Effector A; **PRDM16**, PR/SET domain 16; **SAT**, Subcutaneous adipose tissue; **VAT**, Visceral adipose tissue; **EAT**, Epididymal adipose tissue; **WAT**, White adipose tissue; **BAT**, Brown adipose tissue; **RER**, respiratory exchange ratio; **OCR**, Oxygen consumption rate; **HFD**, High-fat diet; **LFD**, Low-fat diet. **TC**, TotalCholesterol; **TG**, Triglyceride; **LDL**, Low-Density Lipoprotein; **HDL**, High-Density Lipoprotein; **GO**, Gene Ontology; **KEGG**, Kyoto Encyclopedia of Genes and Genomes.
